# Using Geospatial Analysis to Inform Development of a Place-Based Integrated Care Initiative: The Healthy Homes and Neighbourhoods Experience

**DOI:** 10.5334/ijic.5430

**Published:** 2021-06-21

**Authors:** Katherine Todd, John G. Eastwood, Penelope Fotheringham, Jose A. Salinas-Perez, Luis Salvador-Carulla

**Affiliations:** 1 Community Health Services, Sydney Local Health District, Level 9, King George V Building, Missenden Road, Camperdown NSW 2050 Australia; 2 School of Women’s and Children’s Health, The University of New South Wales, Sydney, NSW 2052 Australia; 3 Ingham Institute of Applied Medical Research, Liverpool, NSW Australia; 4 Charles Perkins Centre, Menzies Centre for Health Policy, Discipline of Child and Adolescent Health, and School of Public Health, University of Sydney, Sydney, New South Wales 2006 Australia; 5 Sydney Institute for Women, Children and their Families, Camperdown NSW 2050 Australia; 6 School of Medicine and Public Health, University of Newcastle, Newcastle, NSW 2308 Australia; 7 Centre for Mental Health Research, Research School of Population Health, Australian National University. 63 Eggleston Rd. Acton, ACT 2601 Australia; 8 Department of Quantitative Methods, Universidad Loyola Andalucía. Avenida de las Universidades s/n. 41704 Dos Hermanas, Sevilla, Spain

**Keywords:** social disadvantage, spatial epidemiology, place-based initiatives, planning, geographical analysis

## Abstract

**Introduction:**

Disadvantaged families experience many barriers to accessing health and social care. *The Healthy Homes and Neighbourhoods (HHAN) Integrated Care* Initiative was developed to address these barriers, and ensure families have their complex needs met and are kept safe and connected to society.

**Description:**

A spatial epidemiology approach was taken, as part of the HHAN feasibility phase, to identify the geographical distribution of the “most vulnerable” families in Sydney Local Health District (SLHD). A literature review was conducted to identify indicators of family stress and disadvantage, and cluster and hotspot analyses were undertaken. Hotspots of family stress and disadvantage were mapped for SLHD and used to identify areas for HHAN place-based delivery, and for collaborative co-design.

**Discussion:**

The HHAN initiative called for consideration of context and the undertaking of collaborative design with communities. The spatial analysis provided a more accurate picture of family stress and disadvantage than previously available and provided a tool that could be used during consultation and planning activities.

**Conclusion:**

When planning place-based integrated care initiatives, spatial analysis of small geographic scales can allow identification of areas of concentrated or complex disadvantage that may be masked when analysis is performed on larger areas, allowing for targeted, place-based delivery of programs to those most in need.

## Introduction

We have previously reported in this Journal on the design and proposed evaluation of the *Healthy Homes and Neighbourhoods Integrated Care Initiative* (HHAN). The study reported here was undertaken as part of the previously described feasibility/piloting phase [1]. The focus of the analysis was on identifying the geographical distribution of the most vulnerable families, which would then contribute to identifying suitable locations for the proposed place-based interventions.

Social disadvantage is a multi-dimensional concept, in that it incorporates the ability of people in the community to access resources and participate in the economic and social aspects of society. It arises from a complex interplay between the characteristics of residents living within a community and the effects of the social and environmental context within which they live [2].

**Figure 1:**
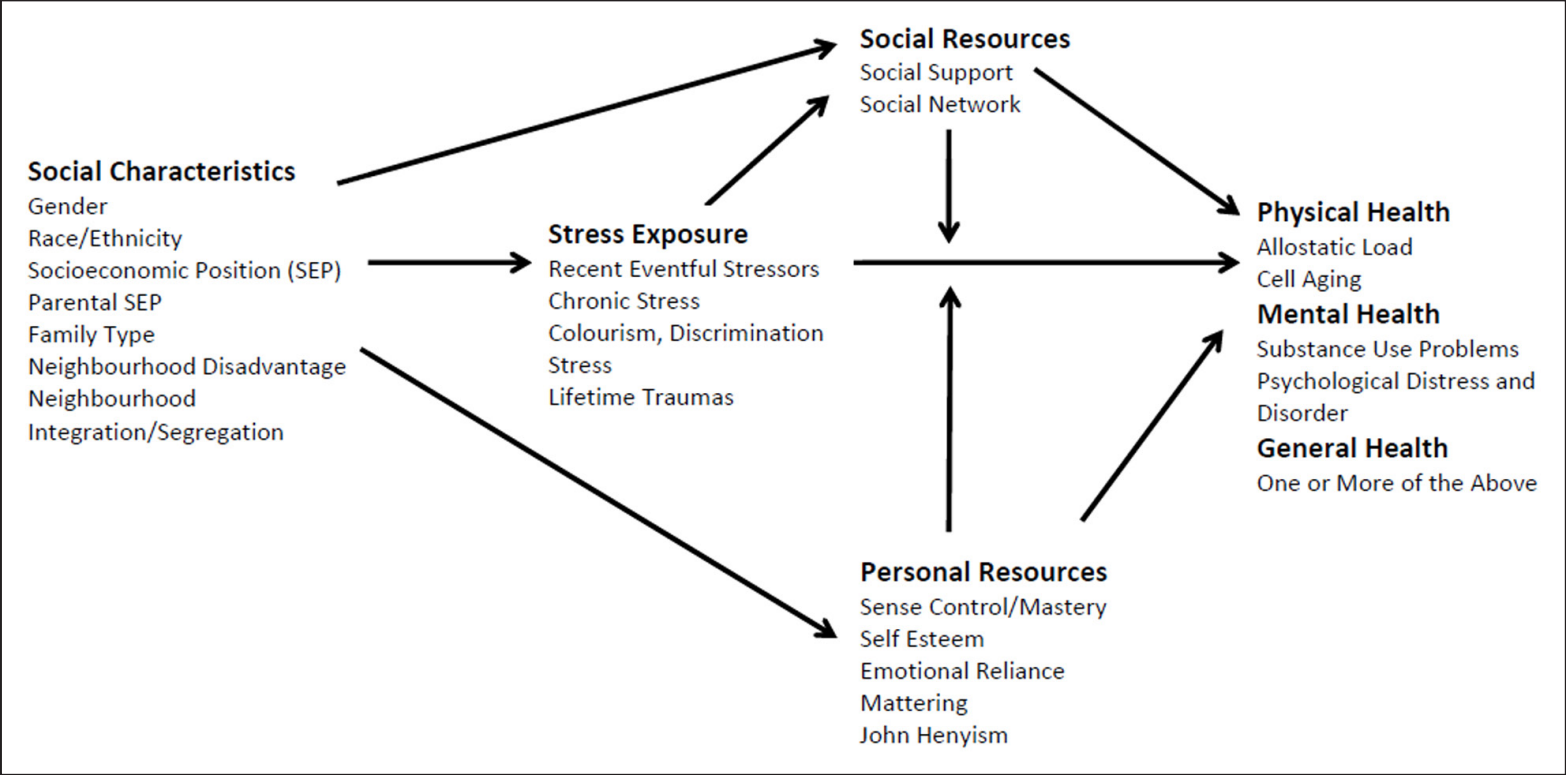
Stress Process Model [4].

The stress process model first described by Pearlin and colleagues [3] explains ways in which social structure influence mental health with a focus on the connection between disadvantaged social status and internal psychopathology. According to stress theory, stressors occur either because of psychological characteristics of individuals or because of environmental factors over which the person has little control (***Figure 1***).

Spatial epidemiology is aimed at identifying patterns in the geographical distribution of health data. Analysis of the geographical distribution of disease and other health outcomes is increasingly used in epidemiology, and conducting spatial analyses allows the utilisation of local information for planning and health policy, whilst ensuring that interventions remain evidence-based [5].

Spatial epidemiology methods are particularly relevant to the analysis of the distribution of disadvantage.

“When social disadvantage becomes entrenched within a limited number of localities a disabling social climate can develop that is more than the sum of individual and household disadvantages, and the prospect is increased of disadvantage being passed from one generation to the next. In such cases general social and economic policies need to be supplemented by **locality specific ones** [6].”

Thus in order to address the problems of disadvantaged areas, policies need to appreciate and understand both the spatial distribution of those disadvantaged areas as well as their character and context [7]. Where an accumulation of problems makes a serious and sustained impact on the wellbeing of residents of a disadvantaged area, locality-specific measures may be needed [8].

Spatial epidemiology is aimed at identifying patterns in the geographical distribution of data and can detect irregularities such as clusters of a disease or disadvantage. Where the data is available and sufficiently robust, analysis of data at small level areas such as suburbs can provide specific information to also inform these place-based initiatives [9] and specific organisational management interventions [10]. The spatial epidemiology study reported here was undertaken in 2015 as part of a translational social epidemiology study [11] to inform the design and implementation of an integrated care initiative for vulnerable families and their children in Sydney Local Health District [12], with a focus on the spatial distribution of perinatal and family social disadvantage and adversity.

Mapping and spatial analytics tools have previously been used to describe the pattern of care provision for mental health disorders in Western Sydney [13], the geospatial distribution of specific mental health conditions (postnatal depressive symptoms in South Western Sydney) [14,15], key health related habits and resource use in both South West and Inner West Sydney (antenatal smoking and time of first antenatal visit) and the implementation and diffusion of the “Access to Allied Psychological Services” program (ATAPS) in the Western Sydney Region, and its relation to social disadvantage [16]. This information was relevant, but not sufficient for actual resource allocation at the local level, where a more granular analysis of small areas where families are experiencing severe adversity is needed.

Such identification of areas of social disadvantage requires the selection of a set of indicators which individually or together could signal the presence of social disadvantage and adversity. Ideally, those indicators should be direct manifestations of disadvantage and adversity, and not be included due to the assumption that concentration of certain groups or membership of certain groups signify disadvantage [8].

Areas where these and other forms of disadvantage and adversity cluster are also areas where confirmed child maltreatment frequently occurs [8]. Vinson (2015) observed that child maltreatment rates were 4.5 times greater in the most disadvantaged areas of New South Wales (NSW) when compared with the rest of the state [8]. The diversity, breadth and interlinkages of markers of disadvantage have been elaborated in an Australian Government-led study into breaking cycles of disadvantage (***Table 1***) [17].

**Table 1:** Key domains and linkages that can both cause and result from disadvantage [17].


DOMAIN	KEY INFLUENCES ON DISADVANTAGE	COMMON INTERLINKAGES

**Primary relationships**	Childhood – Material neglect, financial insecurity and limited resources – Emotional neglect from parents – Family violence – victims/perpetrators/observers – Family relationship breakdown, separation or loss of parent – Negative or absent role modelling – Peer relationships – acceptance/discrimination/bullying Adulthood – Abusive relationships with partners and others – Relationship/family break-ups – Arrival of child and parenting pressures/challenges – Harmful peer relationships	– Alcohol and drug use – Mental health issues – School disruption – Leaving school early – Criminal activity – Difficulty forming relationships – Low self-esteem

**Education**	– Disinterest and disengagement in academia – Low confidence and expectation of achievement – Transience in the school environment/delivery approach – Influential peer relationships – bullying, truancy, illicit behaviours – Service access and delivery within or outside ‘mainstream’	– Lack of employment opportunity – Literacy and numeracy issues – Alcohol/drug use – Criminal activity – Low self-esteem – Social disconnection

**Employment**	– Job loss/redundancy – Long-term unemployment and absence from workforce – Work capacity/capability, confidence and motivation – Discrimination from employers/service providers – Restricted availability and suitability of employment options	– Financial pressure and debt – Health issues – Low self-esteem – Relationship break-down – Homelessness – Loss of skills – Social disconnection

**Health and wellbeing**	– Accident or onset of illness/physical disability – Development of or long-term mental health issues – Trauma and emotional wellbeing – Dependency/substance abuse/addiction (alcohol, drugs, gambling)	– Disruption of education/employment – Long-term unemployment – Relationship break-down – Social disconnection – Other health issues – Alcohol/drug use – Low self-esteem – Incarceration

**Identity**	– Low self-esteem, confidence, sense of purpose – Cultural expectations, behaviours, influence – Discrimination and prejudice	– Lack of employment opportunity – Social disconnection – Low self-esteem – Health issues

**External environment**	– Locality (remoteness and access to community services, relocation/transience) – Housing (condition of housing environment/neighbourhood, homelessness) – Incarceration (reinforcement and normalisation of negative behaviours in institutionalisation, discrimination and segregation following release)	– Lack of employment opportunity – Social disconnection – Risks to safety and wellbeing – Drug/alcohol use – Low self-esteem – Health issues


This innovative approach to understanding the distribution of vulnerable families sought to identify the distribution of those who are not only experiencing exogenous social disadvantage but may also be experiencing adversity and stress from intra-familial features. Our earlier empirical and theoretical studies had identified perinatal adversity related variables implicated in family stress [18,19,20,21]. Based on analysis of that literature and availability of local data, indicators of family disadvantage and adversity were selected for spatial analysis.

### Ethical Approval

Institutional ethics approvals were obtained from both the South Western Sydney Local Health District and the Sydney Local Health District Ethics committees to conduct this data linkage study. Data used for this project were anonymous and no individuals were contacted (Approval numbers HREC: LNR/11/LPOOL/463; SSA: LNRSSA/11/LPOOL/464 & Project No: 11/276 LNR; Protocol No X12-0164 & LNR/12/RPAH/266). The data used for the analysis are accessed in accordance with ethical protocols that only allow unit record information to be released to investigators included in the ethics committee submission for study approvals.

## Description of the Practice

### Overview

This spatial epidemiology study followed a healthcare ecosystem approach [22] and provided a secondary analysis of routinely collected data on maternal and child health information sourced from the SLHD electronic medical record databases together with census data for small areas. The project aimed to identify the geographical distribution of the “most vulnerable” families within SLHD with possible intergenerational cycles of disadvantage and adversity, in order to inform the design of an integrated care intervention that includes place-based initiatives. The process involved:

1. Identifying indicators of disadvantage and mapping them within SLHD
2. Identifying areas where indicators of disadvantage are clustered
3. Analysing potential pockets or “hotspots” of extreme or complex disadvantage via layered analysis of individual indicators of disadvantage.

**Figure 2:**
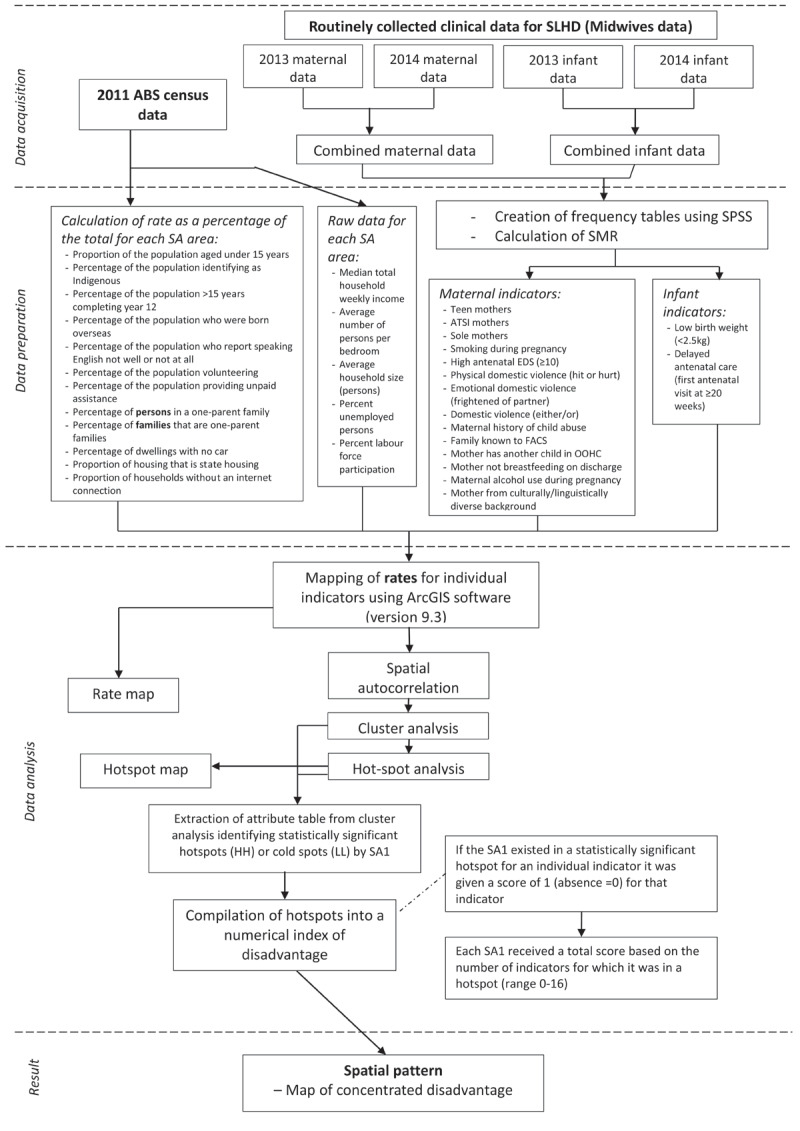
Overview of methods and analysis used.

The full overview of methods is demonstrated in ***Figure 2***.

The knowledge generated in this study was then used to examine the impact of integrated care initiatives on the perinatal adversity as experienced by parents and infants born in SLHD.

### Geographical Location

SLHD provides healthcare to over 580,000 residents of the inner west of Sydney, and has a population density of 4,210 residents per square kilometre. By 2021, the SLHD population is expected to reach 642,000 [12]. SLHD is characterised by socioeconomic diversity, and has pockets of both extreme advantage and extreme disadvantage. SLHD is also an ethnically diverse community, with 51.5 per cent of the population speaking a language other than English at home, and there is a significant proportion of the population who identify as Aboriginal, which is highest in the City of Sydney, Marrickville and Canterbury areas. More than 8,500 babies are born in SLHD annually, representing more than 9 per cent of all births in NSW. In 2014 there were 90,916 infants, children and young people aged 0–15 years living in SLHD [12].

### Data sources

Data for the indicators outlined in ***Table 2*** above were obtained from two main sources:

1. Australian Bureau of Statistics (ABS) [23] 2011 census data
2. Sydney Local Health District linked Maternal and Child Health Data 2013–2014

**Table 2:** Demographic and Perinatal Indicators selected for study.


DEMOGRAPHIC INDICATORS	PERINATAL INDICATORS

– High proportion of the population identifying as Aboriginal or Torres Strait Islander – Low rates of year 12 attainment – Low median weekly household income – High proportion of people reporting speaking English not well or not at all – High proportion of people requiring assistance with activities of daily living (disability) – High proportion of one-parent families – Large proportion of households with no access to a car – Large proportion of housing consisting of state housing – Large proportion of households with no internet access – High rates of unemployment – Low labour force participation rates	– High rates of teen mothers – High rates of sole mothers (pregnant women without partners) – High rates of smoking during pregnancy – High rates of pregnant women with a high antenatal Edinburgh depression score (≥10) – High rates of pregnant women reporting domestic violence (have either been hit or hurt by their partner, or report being frightened of their partner) – High rates of pregnant women reporting a history of child abuse – High rates of families known to Family and Community Services (FACS) – High rates of pregnant women who have other children in out-of-home care – High rates of women who report consuming alcohol during pregnancy – High rates of low-birth-weight (LBW) infants – High rates of pregnant women with delayed antenatal care (first visit at ≥20 weeks)


#### 2011 ABS Census of Population and Housing data

2011 ABS Census of Population and Housing data is available at various levels of scale, known as Statistical Local Areas (SLAs). An SLA is an Australian Standard Geographical Classification (ASGC) defined area which when combined covers the whole of Australia without gaps or overlaps [24]. Statistical Area Level 1 (SA1) is the smallest area of output for the Census of Population and Housing; there are 54,805 SA1s covering the whole of Australia. On average they contain a population of approximately 400 people, and most are designed to be within the population range 200 – 800 people [23].

Data was obtained at the SA1 level from the 2011 ABS Census. Raw numbers as provided by the ABS were used for the following indicators: a) median household weekly income; b) average number of person per bedroom; c) average household size (persons); d) percentage of unemployed persons; and e) percentage of labour force participation.

For the demographic indicators shown in ***Table 2***, a rate was calculated as a percentage of the total for the relevant SA area (i.e. proportion of the total population for that area).

#### Routinely collected maternal and child health data

Data was obtained from the SLHD linked Maternal and Child Health data set from all infants born over the period 01/01/2013 – 31/12/2014. The combined data for the calendar years of 2013 and 2014 consisted of N = 11,536 mothers and N = 11,693 infants. Antenatal data (routinely collected by midwives) were linked using individual identifiers to routinely collected postnatal data relating to perinatal outcomes and maternal health outcomes.

The addresses of de-identified individuals were geocoded and assigned the appropriate SA1 code on construction of the database. Frequency tables with counts of mothers in each SA1 area were constructed using the Statistical Package for the Social Sciences (SPSS). This information was then used to calculate the ratio of observed cases to expected cases, rather than proportion for individual areas given low numbers. The Maternal and Child Health data included indicators of disadvantage or adversity are shown in ***Table 2***.

### Selection of geographic scale

For the purposes of designing a place-based initiative, it was desired to have this index constructed at the smallest geographic area available (in this case, SA1) in order to identify not only the suburbs but the streets or blocks in which concentrated disadvantage was located, in order to provide targeted services to the most vulnerable families.

Determination of an appropriate spatial scale of analysis is heavily influenced by data availability and ethical considerations; it requires an area large enough for sound quantitative analysis but not so large that extensive internal diversity could dilute (and therefore render statistically invisible) any significant spatial concentration of disadvantage [25]; and not so small that it may identify individuals. While in areas of low population density SA1 data may potentially identify individuals, this is the best level of aggregation in urban areas as it provides sufficient granularity to prevent the identification of individual subjects while avoiding the ecological effect in the representation of aggregated data [26].

**Figure 3:**
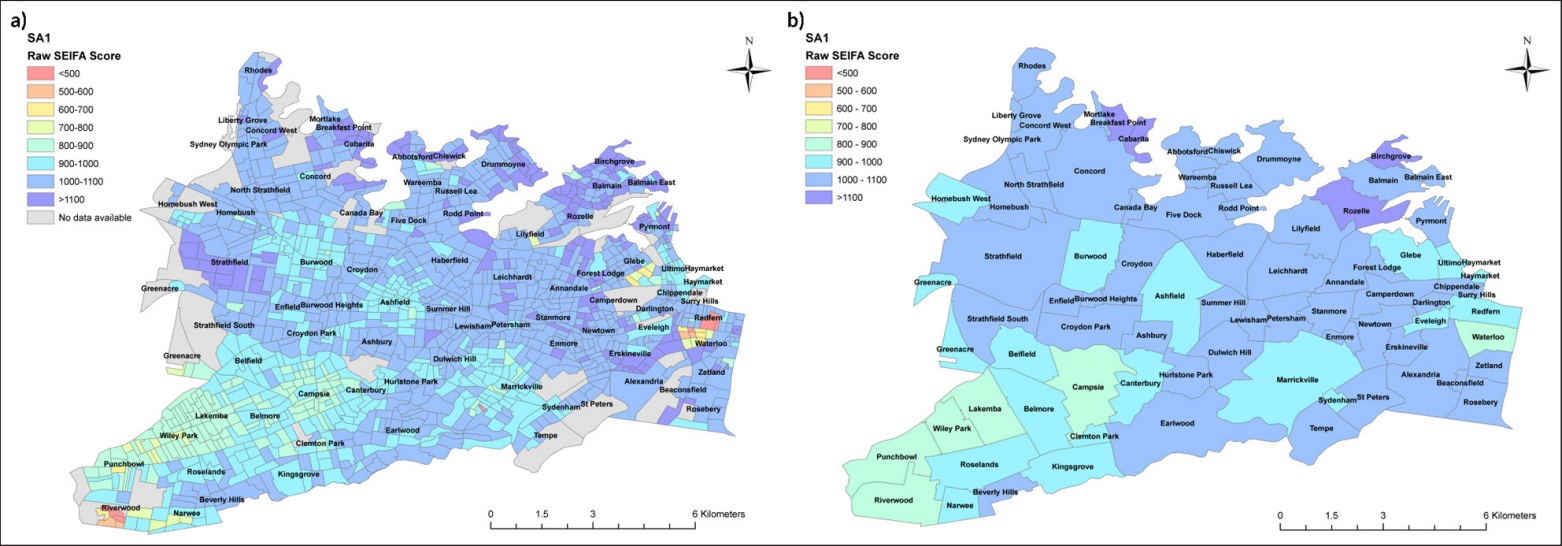
Comparison of SEIFA area if analysis done at **a)** SA1 level, **b)** Suburb level.

This difference is outlined in ***Figure 3***, below, which contrasts the distribution of raw scores of the 2011 Australian Census Index of Relative Socio-economic Disadvantage (IRSD) by SA1 and by suburb. Whilst at the smaller area level pockets of extremely low raw IRSD score (<500) are demonstrated, these smaller areas of concentrated disadvantage are masked when average IRSD score is taken at the suburb level. An example of this can be seen in the suburb of Redfern, where gentrification of a previously disadvantaged inner-city area alongside clustering of long-term public housing has created marked diversity in wealth and social capital across the suburb.

### Data analysis

ArcGIS software (version 9.3) was used to generate maps showing the spatial distribution of each individual indicator. Subsequently the maps were analysed using Moran’s *I* tool and Getis-Ord tool to identify whether data were clustered and if so, where these clusters were located.

The Moran’s *I* function is designed to find clustering using the attribute values (i.e. size and shape of an area) as well as the locations of features. This is typically done with polygons that contain a summary statistics, such as census data or density data. Importantly, the Moran’s *I* function doesn’t identify specific clusters on the map, but rather identifies whether the pattern of values across the study area tends to be clustered, random or dispersed. It does this by comparing the values for neighbouring features – a comparison is made of the differences in values between each pair of neighbours and all the other features in the study area. If the average difference between neighbouring features is less than between all the features, the values are considered clustered [27]. Once the statistical significance of patterns in a dataset is established, it is then possible to pinpoint the locations of clustering patterns [27].

Given a set of weighted features, the Cluster and Outlier Analysis tool identifies clusters of features with values similar in magnitude, as well as spatial outliers. It does this by calculating a Local Moran’s *I* value, a Z score, a p-value, and a code representing the cluster type for each feature [28]. The *Z* score and *p*-value represent the statistical significance of the computed index value. A positive value for *I* indicates that the feature is surrounded by features with similar values (a cluster), whilst a negative value for *I* indicates that the feature is surrounded by features with dissimilar values (an outlier). The COType field distinguishes between a statistically significant (0.05 level) cluster of high values (HH), cluster of low values (LL), outlier in which a high value is surround primarily by low values (HL), and outlier in which a low value is surrounded primarily by high values [12,29].

Hotspot analysis shows graphically where high and low values are clustered [27]. The HotSpot Analysis tool calculates the Getis-Ord’s *Gi* statistic for each feature in a dataset, and resultant (*Z*) score describes where features with either high or low values cluster spatially [30]. The tool works by looking at each feature within the context of neighbouring features – to be a statistically significant hotspot, a feature will have a high value and be surrounded by other features with high values (a cluster). Similarly, a coldspot will have a low value and be surrounded by other features with low values. The local sum for a feature and its neighbours is compared proportionally to the sum of all features; when the local sum is much different than the expected local sum, and that difference is too large to be the result of random chance, a statistically significant *Z* score results and a hotspot (clustering of high values) or coldspot (clustering of low values) occurs [29].

For each individual indicator ArcGIS creates attribute tables that accompany the graphical display; these list the numerical code for each include SA1 area and whether or not that area was in a statistical significant cluster of high values (HH), cluster of low values (LL), outlier in which a high value is surround primarily by low values (HL), and outlier in which a low value is surrounded primarily by high values [12]. These lists were extracted from ArcGIS and used to create a Microsoft Excel spreadsheet combining multiple indicators.

For each indicator clusters of high values surrounded primarily by low values (HL) and low values surrounded primarily by high values [12] were excluded. A decision was made whether a hotspot (i.e. high rates of public housing) or a coldspot (i.e. low rates of year 12 attainment) was the key indicator of disadvantage. Once this decision had been made the opposing value (either LL or HH) was then excluded. Subsequently each time the key cluster (either HH or LL) occurred in an area it received a score of 1.

These scores were totalled to create an overall numerical score for each SA1 area based on the number of times it appeared in a cluster for an indicator of disadvantage. This score was then mapped to create a final map showing the intensity of disadvantage across SLHD (***Figure 5***).

### Findings

**Figure 4:**
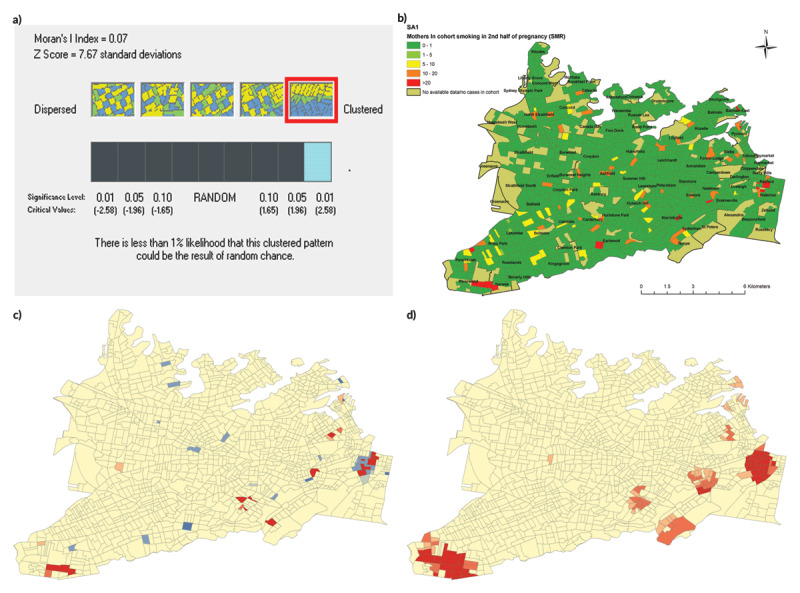
Spatial analysis in ArcGIS of “smoking”.

The outputs of using Moran’s *I* tool and Getis-Ord tool are demonstrated in ***Figure 4*** for an example indicator (*smoking*). The ***Figure 4a***) shows the global Moran’s *I* analysis showing that rates of *smoking* were significantly clustered across SLHD, with a *p*-value of <0.01. ***Figure 4c***) shows the graphical input of the Anselin Local Moran’s *I* analysis, with red areas representing SA1s that had an *I* value of >2.5 standard deviations above the mean for the dataset (i.e. SA1s that are in statistically significant clusters of either high or low values). ***Figure 4d***) shows the results of the Getis-Ord hotspot analysis, which showed hotspots representing areas of high concentration of *smoking* in the eastern side of SLHD.

**Figure 5:**
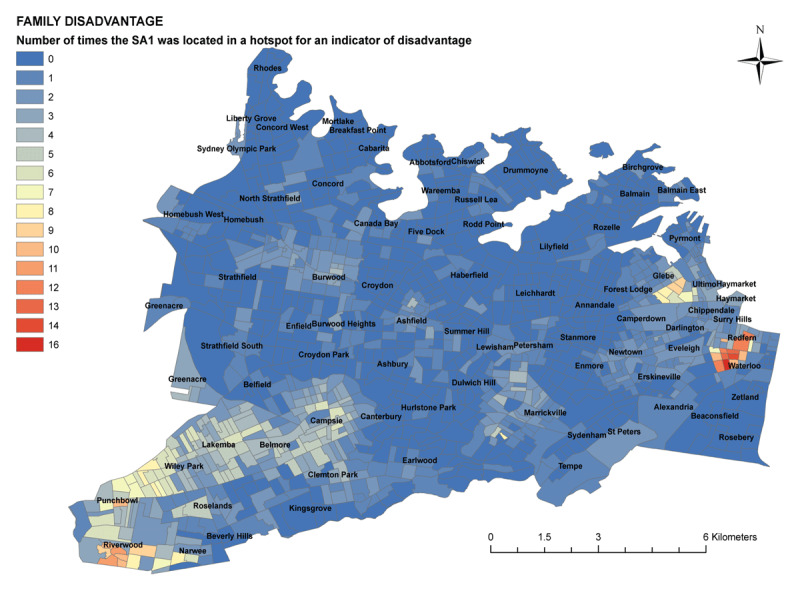
Final Hotspot Analysis of Disadvantage.

The final map showing the intensity of disadvantage across SLHD (***Figure 5***) demonstrated two clear small areas of clustered disadvantage: one in the suburb of Riverwood, and one in the adjacent suburbs of Redfern and Waterloo. In addition, there was an area of less intense, but more widely dispersed, disadvantage in the local government area of Canterbury and including the suburbs of Canterbury, Lakemba, Wiley Park and Punchbowl.

There was a cluster of disadvantage in Redfern and Waterloo, on the eastern border of SLHD, in an area known to have high rates of public housing, unemployment, disability and domestic violence. Several city blocks in this area were in a hotspot of disadvantage at least 11 times, with the area of most intense disadvantage occurring in a hotspot containing 15 of the 22 indicators of disadvantage. Interestingly, despite the fact that Redfern is well-known locally to house residents experiencing significant disadvantage, Waterloo was noted to be more prominently disadvantaged than Redfern in this analysis. This corresponded with the anecdotal reports of staff familiar with the area that families tended to live in public housing in Waterloo, whilst public housing in Redfern was more frequently occupied by single people, often experiencing mental illness and other disabilities.

There was an additional cluster in Riverwood, on the south-western border of Sydney Local Health District and the neighbouring South-Eastern Sydney Local Health District. This area also has a concentration of public housing, is poorly served by public transport, and has a paucity of targeted local health and social services available in part due to the fact that it straddles two health districts.

## Discussion

Family disadvantage and adversity are difficult to define and there has been extensive discussion in the literature about the best framework for analysis. There is no single outcome that can be delineated or measured to quantify disadvantage and approaches for analysis have ranged from economic factors, those relating to access to social capital and the extent of social exclusion relative to health, education, incomes, labour market participation and access to housing [31].

Selection of indicators of disadvantage always involves some element of value judgement [31] and identification of what constitutes an indicator of disadvantage is often contextual and can be controversial. Choosing particular demographic and perinatal indicators for our composite index of family disadvantage and adversity is also subject to these concerns. However in choosing a variety of indicators for this project, we aimed to create a more accurate picture of family disadvantage as it occurs in SLHD. In identifying areas of disadvantage, the aim is to facilitate appropriate service deployment and enable appropriate program evaluation. It is important that in constructing maps at small scale, privacy and the potentially for identification of individuals or communities is considered [32]. This includes the potential for maps in and of themselves to create stigma for places or the communities living in those places. Although there is little in the peer-reviewed literature on the potential links between maps and creation or perpetuation of community stigma, the need for careful consideration of how maps are used has been described in other settings – for example, in 2014 Timo Luge from Médecins Sans Frontières described the measures taken to carefully maintain the security of maps used in managing the Ebola in Guinea due to the stigma associated with Ebola [33]. The concern that this analysis can identify and stigmatise those living in the identified areas is valid and should always be considered by researchers and policy makers; however in our view is balanced by the purpose of the identification and the benefits that can be derived from delivering place-based interventions.

As previously outlined, indicators of disadvantage and adversity are often linked with a cause and effect cycle that are interrelated and have the effect of perpetuating disadvantage. They occur across multiple life domains, including health, wellbeing, relationships, education and housing. As Vinson (2009) outlines, problems of entrenched disadvantage occur with and as a result of limited education, unemployment, poor health, disabilities, limited income, and participation in crime.

Addressing problems of entrenched disadvantage is challenging, given that there are such diverse, interlinked causes. Complex relationships exist between cause and effect, and often outcomes are unpredictable despite a good understanding of the relevant causative factors. Due to these factors solving the problem of disadvantage is beyond the capability of a single player, organisation or level of government [17].

Multi-layer geospatial mapping assisted us in this case to identify where the areas of entrenched disadvantage existed in the community, allowing us to target particular locations. Understanding where disadvantage exists within a community has potential to be an effective strategy to address the effects of disadvantage by creating place based programs building on local expertise whilst working in partnership with members of disadvantaged communities [9].

The HHAN research and evaluation protocol [1] called for consideration of context and the undertaking of collaborative design with communities and their members. The spatial analysis provided a more accurate picture of family stress and disadvantage than previously available to SLHD and provided a tool that was then used during consultation and planning activities. The final map was heavily informed by indicators taken from the local maternal and child health linked data-set. The indicators used, for example interpersonal violence in pregnancy and depressive symptoms, were directly relevant to the HHAN model of care. Similar maps had been produced using census data but the analysis undertaken here was considered more “real” to the HHAN collaboration. The detail available in the map also enabled practitioners to identify city blocks with high levels of family stress.

Importantly, the final map played an important role in ensuring that the needs of the Riverwood community were addressed. That disadvantaged community is on the border of three Local Health Districts and the needs of the community had not previously been prominent in planning undertaken by metropolitan health and social services. The map was consequently able to be used at metropolitan and state-level interagency planning meetings as an illustrative tool and “call to action” for this vulnerable community, and has contributed to the narrative concerning concentrated family disadvantage.

### Strengths and limitations

Overall, this process was extremely useful in providing evidence to inform the planning of pilot sites for the rollout of the Healthy Homes and Neighbourhoods project, and particularly identifying a location by which to house the “in community” portion of the project. However, the process taken here involves secondary use of data collected for administrative purposes, and thus the quality of the data could be variable. In particular, the quality of data in the Midwives dataset could vary from location to location, and blank data fields could mean that areas where some indicators are clustering could have been obscured due to incomplete data collection. In addition, given the small areas used, there were small numbers present which may have exaggerated potential effects. It would be useful to repeat this analysis at time intervals using updated datasets to see if any changes to the geospatial distribution are occurring over time.

### Significance for Integrating Care

Health need is not evenly distributed within society and it is increasingly being recognised that the social needs of patients impact significantly on their health, well-being and use of health services. Social epidemiology is the study of the distribution of advantages and disadvantages experienced by people in society and the consequent impact on the distribution of health and disease. The tools used by social epidemiologists such as those described in this manuscript have significant utility when applied to the planning, implementation and evaluation of integrated health and social care interventions. The HHAN collaborative design process [32] drew on the findings of earlier social epidemiology studies of maternal depression [14] and the findings of a situation analysis [33] that indicated that place of residence matters to well-being. Consequently the HHAN design called for two place-based demonstration initiatives within an inner-city metropolitian context. The study described here not only assisted in identifying priority localities for the HHAN integrated care initiation but has continued to inform other District health service planning processes.

### Lessons learned

- Routinely collected medical record data can be used to identify families and communities with high levels of disadvantage, adversity and stress
- Small area analysis can be undertaken while protecting the privacy of individuals
- Small area analysis can identify previously unrecognised small areas of extreme disadvantage
- The comprehensive nature of routinely collected health data provides valuable information to inform “whole of system” planning

## Conclusions

Spatial analysis of health and demographic data at small geographic scales can allow identification of hotspots of concentrated or complex disadvantage that may be masked when analysis is performed on larger areas. This identification can then allow targeting of the most disadvantaged and most vulnerable families in the community, and delivery of place based initiatives, health initiatives and services that are tailored to local need.
